# Promoting health equity through cross-sector strategies: the integration of communication public health

**DOI:** 10.3389/fpubh.2025.1576973

**Published:** 2025-05-19

**Authors:** Leslie Lopez, Sarah Warren, Teresa Anthony, Laura Coco

**Affiliations:** ^1^LSU Health Sciences Center New Orleans, Louisiana State University, New Orleans, LA, United States; ^2^School of Communication Sciences and Disorders, University of Memphis, Memphis, TN, United States; ^3^College of Public Health, University of South Florida, Tampa, FL, United States; ^4^School of Speech, Language, and Hearing Sciences, San Diego State University, San Diego, CA, United States

**Keywords:** communication health, health equity, social determinants, speech-language pathology, audiology

## Abstract

Communication is a fundamental human experience at both inter-and intrapersonal levels, deeply intertwined with societal development. Because of the integral role of communication in society, challenges in communication—whether due to differences, disorders, or environmental factors—can create significant barriers to full societal participation. Rather than viewing these challenges solely through the lens of “disability,” recognizing *communication health* as a distinct and essential aspect of public health is crucial for addressing these barriers and advancing health equity. This viewpoint introduces the concept of *Communication Public Health*, advocating for an integrated approach that unites the fields of communication sciences and disorders and public health to address *communication health* as a social determinant of health. It presents a conceptual framework that emphasizes collaboration across the education, employment, housing, and community sectors, highlighting the importance of perceiving communication challenges as modifiable barriers to full societal participation. It aims to offer insight and debate, and mobilize public health professionals to recognize and prioritize *communication health*, offering a comprehensive approach that considers both individual and societal needs. By establishing *Communication Public Health* as a new and distinct entity within public health infrastructure, we can better address the full spectrum of communication needs, from individuals with communication disorders and differences to those with healthy communication abilities. This perspective article format allows us to synthesize underexamined intersections between communication sciences and public health, and to advocate for an emerging framework in the absence of an established empirical evidence base.

## Introduction

Reducing health inequalities is a global priority that requires action at a societal level, as health is profoundly influenced by drivers such as education, housing, access to healthcare, employment, income, food, social cohesion, and social status (1, 2). These factors collectively shape health and health inequalities, as access to services and opportunities varies widely within and across countries (2, 3). The perspective format is intentionally selected to stimulate interdisciplinary discussion and action around the integration of *communication health* into public health systems, a topic that lacks a consolidated empirical foundation but presents pressing conceptual and practical implications (4). This commentary highlights the role of *communication health* as a critical yet underrecognized determinant within this framework. By integrating *communication health* into broader cross-sector strategies, including interventions and policies targeting education and employment, *Communication Public Health* offers a transformative approach to advancing health equity.

Communication is an essential human experience at the inter-and intrapersonal levels, deeply embedded in society, with “communication” and “community” sharing similar roots (5). The exchange of information, ideas, and emotions has been vital to the enhancement of individual quality of life, the formation of communities, and the advancement of civilization. Communication, encompassing both comprehension and expression and involving speech, language, hearing, and alternative modalities, is integral to every aspect of daily life for all individuals. Speaking, listening, signing, reading, writing, and other forms of communication are crucial for psychosocial development, education, economic transactions, entertainment, community engagement, health care, and overall human experience (6).

Communication extends beyond words alone, which account for only 7% of communication, and includes all messages expressed, even nonverbal communicative acts such as gesturing, eye gaze, intonation, and stress (6). On an individual level, communication begins by meeting basic needs and quickly evolves to become fundamental in building human connections and relationships. Audiologists (AuDs), speech-language pathologists (SLPs), and other speech, language, hearing, and related professionals (SLHP+) play a vital role in supporting individuals with communication limitations, enhancing their abilities to connect with others and participate in broader societal interactions. However, compared to physicians and nurses, SLHP+ are often less recognized in international health systems, despite their critical role in promoting *communication health* and supporting broader health outcomes (7–9). In many healthcare contexts, social and health professions are predominantly traced back to the medical model, limiting the visibility and integration of allied health fields, including those addressing speech, language, and hearing, in public health infrastructures. At a societal level, communication serves as the backbone for community cohesion, public health initiatives, and the equitable dissemination of information. Since individuals and society are interlinked, communication plays a pivotal role in the health and wellbeing of individuals and populations (10). Barriers to effective communication, whether due to language, development, or circumstance, may hinder societal participation. Removing these obstacles is essential for building a more connected and just society, where communication remains a tool for inclusiveness, driving social unity and collective progress. Social cohesion and inclusion are fundamental pillars for advancing *communication health* at the societal level. Collaborative initiatives that bring together public institutions, private organizations, and non-governmental organizations (NGOs) are critical in developing and testing innovative approaches that support communication equity. For example, NGO-led programs, such as the use of art therapy in Alzheimer’s disease care, demonstrate how intersectoral partnerships can foster inclusive environments that promote social connection and communication participation among vulnerable populations (11). Embedding similar collaborative models within *Communication Public Health* initiatives can help address communication barriers while strengthening community resilience and wellbeing.

The concept of disability has a long and complicated history, the effects of which still echo throughout society today (12, 13). Challenges in communication can cause individuals to face barriers fully participating in society; however, these challenges can be addressed and thereby mitigated, allowing individuals to engage more fully and effectively in their communities. Working within both medical and social models, SLHP+ aim to empower individuals with communication challenges by helping them apply communication strategies and participate more fully in their communities. Additionally, SLHP+ collaborate with community members to train them in supportive communication approaches, reflecting the core purpose of these professions—to facilitate communication and foster inclusion. In addition to the medical and social models, the biopsychosocial model serves as a prevailing framework that integrates biological, psychological, and social factors in understanding health. While *communication health* is influenced by these same determinants, we propose *Communication Public Health* as a way to operationalize these concepts specifically for communication equity, emphasizing its systemic and societal implications, which remain underdeveloped in current public health strategies. Recognizing *communication health* as a distinct and essential aspect of public health infrastructure, rather than merely categorizing it under “disability,” is key to addressing barriers and promoting health equity. Communication differences, such as the various dialects spoken across different regions, the use of sign language by members of the Deaf community, and variations in speech patterns among stutterers, should not be viewed as disorders. These communication differences reflect the rich diversity of human communication and are integral to cultural and individual identity.

*Communication Public Health* differs fundamentally from traditional disability models by shifting the focus from categorizing individuals based on impairments to addressing communication barriers as modifiable social determinants of health (SDOH). While disability frameworks often emphasize accommodations and support for individuals identified as having specific impairments, *Communication Public Health* adopts a proactive and inclusive approach aimed at creating environments that support effective communication for all, regardless of ability (14). This perspective also contrasts with health communication models, which primarily address the dissemination of health information rather than the capacity of individuals to communicate and engage within their communities (15). These theoretical distinctions provide the foundation for examining how systematic structures such as education, healthcare, and employment can better respond to communication needs across the lifespan. Positioning *communication health* as a population-level priority allows *Communication Public Health* to transcend the limitations of both disability and health communication frameworks, advocating for interventions that promote communication equity as a means of achieving broader health and social outcomes. By prioritizing *communication health*, we can create more inclusive environments that empower all individuals to engage meaningfully in all aspects of life. Recognizing the relevance of these models allows for targeted structural changes that reinforce *communication health* as both a clinical and population-level concern. To achieve this, it is essential to establish *Communication Public Health* as a new and distinct entity within public health infrastructure. *Communication Public Health* is more than just language concordance – it captures the full spectrum of communication needs, inclusive of both individuals with communication disorders and those with healthy communication abilities. This multidimensional approach allows for targeted interventions and policies and fosters a collective responsibility to address communication barriers while also promoting effective and inclusive communication for all. By adopting *Communication Public Health*, we can ensure that public health initiatives are comprehensive, ultimately leading to a healthier and more equitable society.

**Figure 1:**
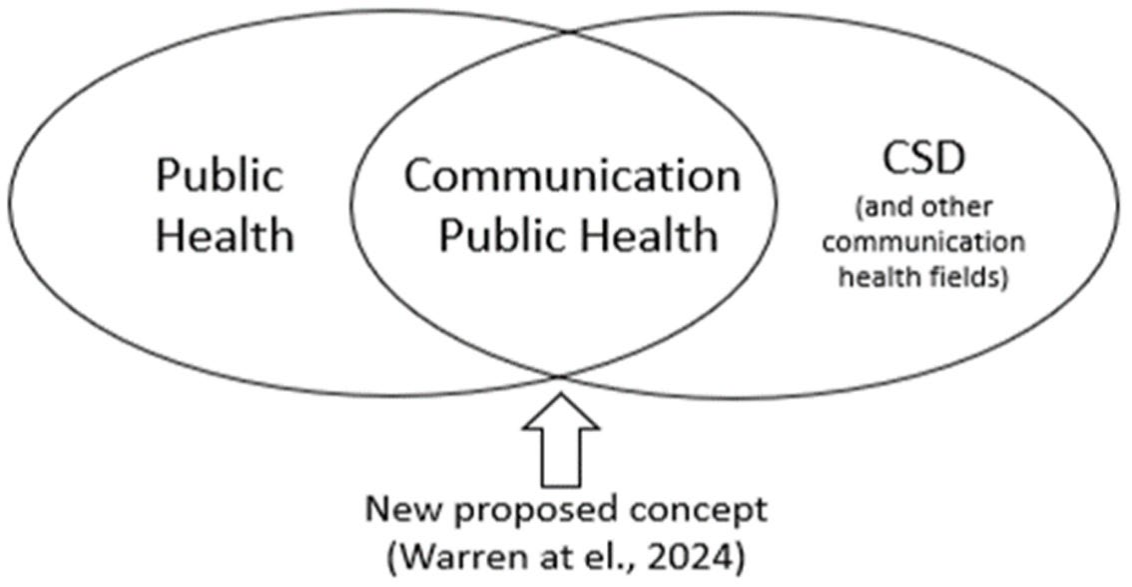
Schematic of the relationship between public health and communication sciences and disorders.

This viewpoint, as a counterpart to Warren, et al. (2024), seeks to appeal to both communication sciences and disorders (CSD) and public health professionals to align their fields in the formalization of *Communication Public Health* (16). It aims to provoke transformational thinking at a high level and mobilize public health professionals to recognize and prioritize *communication health*. This reconceptualized design of *Communication Public Health* (Figure 1) targets public health audiences to engage in a collaborative bridge between public health and CSD, and charges public health professionals to incorporate *communication health* into broader health promotion and disease prevention strategies. Speech, language, and hearing sciences function in the CSD domain, engaging both clinicians promoting *communication health* and researchers trained in other areas, including linguistics, biology, psychology, and public health. Clinical experts in *communication health* include SLPs and AuDs and are interprofessionally connected with a variety of additional disciplines such as otolaryngologists, pediatricians, early interventionists, and non-health professionals such as teachers and paraprofessionals. The scope of practice for SLPs can be found at: https://www.asha.org/policy/sp2016-00343/ and the scope of practice for AuDs can be found at: https://www.asha.org/policy/sp2018-00353/ (17, 18).

This commentary addresses a significant gap in public health literature. It highlights that terms such as, “speech-language pathology,” “audiology,” and “communication disorders” have rarely appeared in public health-focused periodicals since the 1950s, marking a noticeable disparity in the recognition and integration of *communication health* within public health discourse. Furthermore, no systematic reviews exist on this topic, marking this viewpoint as a pioneering effort to bridge CSD and public health and emphasize the importance of *communication health* in achieving health equity and improving public health outcomes. Due to the limited literature on the intersection of CSD and public health, this viewpoint draws on publicly available professional practice standards, existing national and international publications, and the authors’ experiences as practitioners, clinical educators, healthcare operations leaders and administrators, and researchers with expertise in both CSD and public health. This commentary emphasizes the design and delivery of interventions that address the wider determinants of health while recognizing the drivers of inequalities across the life course. It proposes actionable pathways to embed *communication health* within cross-sector decision-making processes, fostering inclusive environments, and promoting equitable health outcomes for all. By outlining practical applications across education, employment, housing, and community sectors, this commentary demonstrates how Communication Public Health can inform concrete scalable strategies to promote inclusion and equity. The examples provided explain how theoretical frameworks can be translated into systems-level public health action.

## Communication disorders in the population

Speech, language, and hearing disorders affect individuals across all ages and regions of the world, yet the prevalence, early detection, and service availability vary greatly by country income level. According to the World Health Organization (WHO), over 1.5 billion people globally experience some degree of hearing loss, with 430 million requiring rehabilitation services, with most living in low-and middle-income countries (LMICs) (3). Many LMICs lack formalized newborn hearing screening programs, public awareness and education campaigns, and accessible clinical pathways for early identification and intervention (3). Speech and language disorders also remain underdiagnosed in these settings due to limited specialist availability and competing health priorities (19).

According to the Royal College of Speech and Language Therapists (RCSLT), approximately 20% of the UK population may experience some communication difficulties at some point in their lives (20). Speech Pathology Australia reports that roughly one in seven Australians experiences some form of communication difficulty (21). In the US, the National Health Interview Survey (NHIS) provides the most comprehensive data available on communication disorders (22). In the most recent report, approximately 6% of adults aged 18 and over reported difficulty communicating, and 15% reported difficulty hearing (22). The National Institute on Deafness and Other Communicative Disorders (NIDCD) estimates that approximately 7.7% of children present with speech disorders and 7.6% of children are living with language disorders (23). The RCSLT reports similar prevalence, with approximately 7.5% of children having a developmental language disorder (DLD), and it is reported that approximately 11% of Australian children present with communication difficulties (20, 21). Often, these disorders have long-lasting effects that persist into adulthood. The incidence of permanent childhood hearing loss is 1.1/1000 and increases to 5.9/1000 for newborns in the neonatal intensive care unit in highly developed countries (e.g., UK, US, Germany) (23). Prevalence of hearing loss varies with age, with rates as high as 13% among adolescents, 3% for adults in their 20s, and increasing to above 50% for adults aged 60 and older (24).

The aging global population has contributed to a significant rise in adult-onset communication disorders, including age-related hearing loss, post-stroke aphasia, and cognitive-communication difficulties related to Alzheimer’s disease and other dementias (19, 20, 22). In many cases, older adults face unique barriers to care due to limited access to hearing technology, low uptake of rehabilitation services, and complexity navigating digital health systems, with isolation confounding these conditions (3). While early detection and intervention for speech, language, and hearing disorders is successful in promoting *communication health* across the lifespan, disparities exist in communication health care utilization, particularly for minority, low-income, and underserved populations. For example, common barriers to equity in pediatric speech, language, and hearing health care include socioeconomic status, poverty, and caregiver education level; postpartum depression; rurality and distance to diagnostic centers; private or public insurance coverage for health care services; access to qualified communication healthcare professionals; and cultural and linguistic differences (25, 26). Similarly, adults with communication disorders also face significant disparities, including lack of access to hearing aids and cochlear implantation, and gaps in communication support for individuals with neurogenerative conditions such as Parkinsons’s disease or Alzheimer’s dementia (27–29). Additionally, adults recovering from traumatic brain injury may encounter challenges in receiving comprehensive cognitive-communication rehabilitation due to health insurance coverage and/or benefit limitations or insufficient interprofessional care coordination (30). Addressing these barriers is essential to promote equitable communication health care across the lifespan.

The prioritization of *communication health* is evident in various national and international programs and organizations, such as the World Health Organization (WHO), International Association of Communication Sciences and Disorders (IALP), the International Society of Audiology, the European Speech and Language Therapy Association (ESLA), the European Federation of Audiology Societies, and Healthy People 2030 (31). Despite the visibility of global initiatives, the intersection of CSD and public health remains largely underrecognized. Several public health frameworks include SDOH, aligning with the principles of *Communication Public Health*, yet they often do not explicitly recognize “*communication health*” as a distinct determinant. For instance, the WHO’s SDOH framework considers factors such as socioeconomic status, education, and the physical environment, but does not specifically include *communication health* in its determinants (32). Similarly, the Centers for Disease Control and Prevention’s (CDC) Healthy People 2030 initiative emphasizes health literacy and effective health communication strategies, focusing on the dissemination and comprehension of health information and the communication exchanges between patient and provider, but does not explicitly address the foundational aspects of *communication health*, such as speech, language, and hearing (31). Organizational Health Literacy (OHL) offers a complementary approach by encouraging institutions to actively reduce access barriers through user-centered design, including individuals with communication disorders (33). Recent applications of OHL in multisectoral health reform efforts have shown promise in developing equity through user-centered service design for populations with communication difficulties (34). By integrating *communication health* into these established frameworks, public health initiatives can more comprehensively address the multifaceted needs of diverse populations, promoting greater health equity and wellbeing.

Guaranteeing equitable access to communication is fundamental for fostering inclusion, thriving in all aspects of daily living, and achieving one’s desired quality of life. In response, this realization has spurred a growing awareness within the field of CSD to adapt to and align with the evolving health care landscape. The field of CSD has broadened its focus to include public health initiatives that emphasize promoting population health, preventing diseases, and addressing social and environmental factors influencing *communication health* equity (17, 18). Mirroring this shift, public health terminology and the interconnected nature of language and public health have garnered recent attention (35, 36). This presents a crucial opportunity to better understand and define the relationship between CSD and public health. In recognition of this opportunity, the authors published a tutorial to CSD audiences clarifying public health terminology, providing context for existing public health efforts within CSD, and defining two new terms: *communication health* and *Communication Public Health* (16).

## Defining communication public health

**Figure 2:**
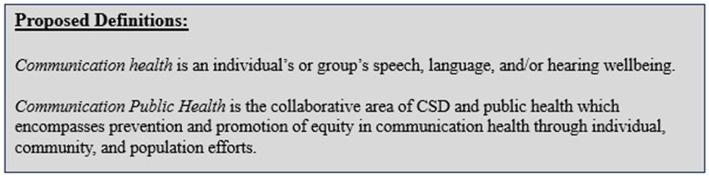
Proposed definitions for communication health and communication public health.

**Figure 3:**
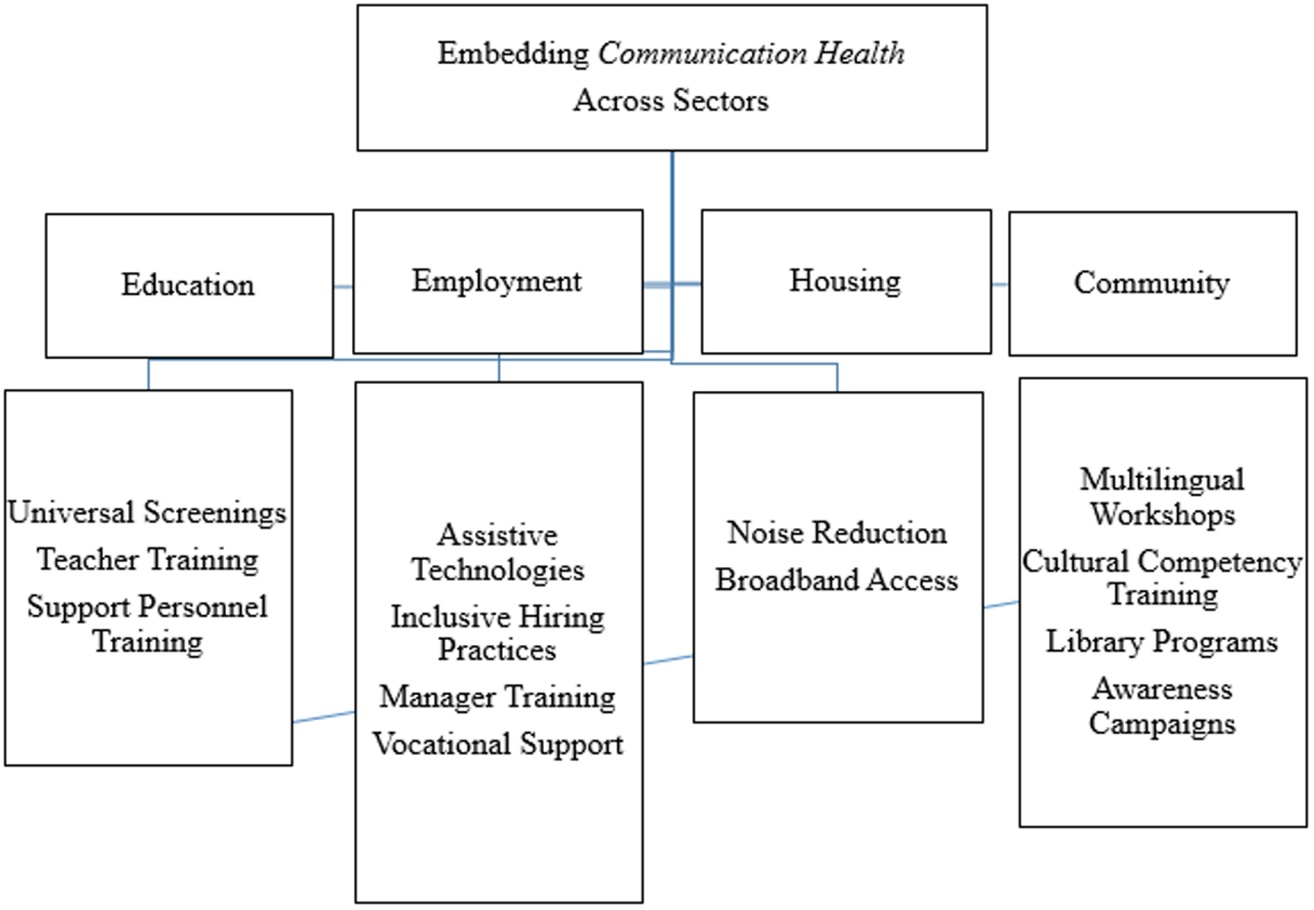
Application of communication public health in broader population health initiatives.

In discussing *Communication Public Health*, it is important to first introduce and distinguish the definition of *communication health*. In Warren et al., 2024, we defined *communication health* as an individual’s or group’s collective speech, language, and/or hearing health and wellbeing (Figure 2) (16). *Communication health* is considered within the context of a person’s preferred language and mode of communication and can be applied from the individual to the population level. For example, a primary care physician (PCP) may screen an individual’s *communication health* by using a questionnaire to identify the individual’s perceived speech, language, and/or hearing status. Additionally, this PCP may express concern about the collective *communication health* of an underserved population within a nearby rural community. This concern may motivate the physician to engage in interprofessional relationships which enable patient-and family-centered care. *Communication health* is proposed as a way of thinking beyond speech, language, and hearing that extends further than discrete biological processes and the medical approach to address communication needs to also consider social factors. Optimal *communication health* is achieved through preventing communication disorders and promoting effective communication at the individual-, community-and societal levels (Figure 3).

One practical application of *communication health* is the implementation of telehealth services that accommodate diverse communication needs, such as offering captioning or sign language interpretation for virtual healthcare appointments, and interprofessional clinical management of patients with voice, swallowing, and upper airway disorders (37, 38). Another example is school-based screening programs that assess students’ *communication health* to identify those at risk for language or hearing difficulties. Additionally, in community health settings, accessible communication resources may be integrated into stroke rehabilitation programs to support individuals with aphasia, promoting timely and appropriate follow-up care and community reintegration support. Similarly, hearing health campaigns aimed at older adults can include routine hearing screenings in primary care settings, along with partnerships between healthcare providers and AuDs to facilitate hearing evaluations, hearing aid fitting, and auditory training. These efforts are supported at the systems level by federal and state policies that mandate access to telehealth services and hearing health care for all individuals, no matter their age or health insurer. These initiatives demonstrate how *communication health* transcends traditional health literacy or disability models by proactively identifying and addressing communication barriers within community settings.

It is imperative to note that *communication health* differs from health communication, which focuses on how health messages are disseminated to, and consumed by, the public. While health communication aims to inform and educate individuals about health-related topics, *communication health* emphasizes the fundamental ability of individuals to express themselves, understand information, and engage meaningfully with others. *Communication health* is more than health literacy. Recognizing this distinction is essential for developing comprehensive strategies that address both the dissemination of health information, and the broader communication needs of diverse populations. By integrating *communication health* into public health initiatives and infrastructure, we can create more inclusive and effective approaches to promoting wellbeing for all.

*Communication Public Health* is proposed as a collaborative area of CSD and public health which encompasses prevention and promotion of equity in *communication health* through individual, community, and population efforts. In *Communication Public Health*, professionals collaborate with diverse and engaging partner teams, including government agencies, community organizations, healthcare providers, and public health departments to accurately identify those at risk, educate the public, advocate for those in need, and create environments that promote and support *communication health* in the population. The goal of *Communication Public Health* is to improve *communication health* outcomes, while simultaneously reducing *communication health* disparities.

*Communication Public Health* acknowledges that interventions informed by social and behavioral science theories offer the most effective avenue for enhancing *communication health* (39). Merging these distinct elements allows for a more unified, comprehensive perspective to examine, explain, and guide clinical and research endeavors. By applying an integrated perspective, *Communication Public Health* encourages a comprehensive understanding of communication that extends beyond individual-level processes, fostering a more holistic approach to addressing communication disorders and differences and promoting effective communication, while simultaneously fostering equity.

## Application of *communication public health* in broader population health initiatives

The scope of *Communication Public Health* extends beyond addressing primary speech, language, and hearing disorders. Factors which affect *communication health* may co-occur with primary and secondary speech, language, and hearing disorders. For example, the promotion of smoking cessation not only reduces the risk of various chronic health conditions and risk for disease but also indirectly decreases the likelihood of stroke, consequently reducing the risk of aphasia, balance disorders that result in falls, and other concomitant communication disorders (40). Acknowledging the role of communication, particularly in the context of language-based learning disorders, is important to understanding the broader implications for public health. Children with language-based learning and auditory processing disorders are at an increased risk for academic difficulties, social challenges, and behavioral issues, which can contribute to a higher likelihood of engaging in criminal behavior and eventual incarceration (41). Addressing language functioning is a critical aspect of *Communication Public Health*, as early intervention and support can significantly reduce the risk of future criminal justice involvement and promote healthier, more equitable outcomes. Likewise, advocating for widespread vaccination prevents prenatal infections known to contribute to congenital hearing loss and cognitive impairment affecting communication development (42). For example, families and practitioners with first-hand experience of childhood sensorineural hearing loss resulting from congenital Cytomegalovirus (cCMV) are key drivers of policy efforts to instate a universal cCMV screening at birth (43). *Communication Public Health* initiatives can promote the integration of aphasia-friendly communication strategies within community health services, too, including training healthcare staff to use visual aids and supported conversation strategies (44). These examples emphasize the permeating nature of objectives in *Communication Public Health*, prompting collaborative ventures across diverse professional domains beyond CSD. This beyond-the-individual perspective is an important element of population health approaches (45). These examples represent a few of the many ways *Communication Public Health* can be applied, highlighting its broad and multifaceted nature. These examples are not exhaustive, however, and do not include the full range of possibilities within this emerging area.

## Actionable pathways to embed *communication health*

Effective implementation of *Communication Public Health* requires collaboration among professionals and sectors from multiple disciplines, including, but not limited to, public health experts, epidemiologists, policymakers, technology companies, and SLPs, AuDs, and SLHP+. Epidemiologists can play a vital role by analyzing public health data to identify populations at high risk for *communication health* disparities. For example, epidemiologists may examine correlations between socioeconomic status, geographic location, and prevalence of untreated hearing loss, traumatic brain injuries, or language-based learning disabilities. This data-driven approach helps to identify communities where *communication health* interventions are most needed. Policymakers, in turn, may use these findings to advocate for funding and policy changes that address barriers to *communication health*, such as inadequate access to audiological care or language services in healthcare settings.

Technology companies, particularly those involved in telehealth, hearing health care, and digital health platforms, may collaborate with public health experts and SLHP+ to develop accessible interfaces and features tailored to users with communication challenges. This may include real-time captioning, automated speech recognition, seamless Bluetooth connections for hearing technology use, or options for sign language interpretation in all community settings. Broadband providers also have a crucial role, as reliable and affordable internet access is foundational for implementing telehealth solutions, especially in rural or underserved areas where *communication health* disparities are most pronounced (31).

To illustrate this collaborative approach, consider a community-level initiative to improve healthcare accessibility for individuals who use augmentative and alternative communication (AAC). Public health professionals, SLPs, AuDs, and SLHP+ would work together to develop protocols for healthcare staff to communicate effectively with individuals who use AAC including training on device operation, interpreting synthesized speech, and using visual symbols. Technology companies would also be a key interprofessional team member to confirm that healthcare applications and telehealth platforms are compatible with common AAC systems, allowing seamless integration for patients who use voice output communication aids or text-based devices. Broadband providers would further support these efforts by guaranteeing high-speed internet access in healthcare settings, enabling real-time communication between patients using AAC and their healthcare providers. This type of initiative would reduce communication barriers between providers and patients in medical environments and provide guidance on how multi-sector interprofessional collaboration can address communication disability as a determinant of health and promote more equitable healthcare outcomes.

### Education

Embedding communication health into cross-sector collaboration requires targeted strategies that address specific societal determinants. In education, integrating speech-language-hearing services into school-based health programs allows for early identification and intervention for communication challenges, particularly in underserved areas. While many countries already provide speech-language services within schools, an innovative and impactful approach involves pairing schools with public health agencies to enhance service delivery and address broader determinants of *communication health*. One way to achieve this collaboration is by establishing formal partnerships between school districts and local public health departments to develop comprehensive *communication health* programs. These partnerships can leverage epidemiological data to identify communities with the highest prevalence of communication challenges, guiding resource allocation and targeted interventions. Public health agencies can also support schools by training personnel on *communication health* literacy, equipping teachers and staff with the knowledge to recognize potential communication difficulties and understand their impact on academic and social success. Additionally, public health professionals can assist in developing standardized screening protocols and data management systems that integrate with existing school health records, streamlining the process of identifying students at risk. By pooling resources, schools and public health agencies can coordinate outreach programs to educate families about *communication health* and connect them to community-based services, reducing disparities in access to care. This collaboration may also support the development of multidisciplinary teams within schools, including not only SLPs and AuDs, but also public health nurses, community health workers, and social workers, to address *communication health* as part of a holistic approach to student wellbeing.

### Employment

In employment, workforce development initiatives can prioritize accessible workplace communication by promoting assistive technologies such as speech-to-text software and captioning services. Employers can also collaborate with *communication health* professionals to develop training modules for supervisors to support a variety of communication needs. Inclusive hiring practices, such as ensuring interview processes accommodate individuals with speech, language, or hearing differences, can further enhance equity in the workplace. Programs that pair businesses with vocational rehabilitation services can help individuals with communication challenges transition into employment with the necessary support structures. Building on these foundational strategies, innovative cross-sector partnerships can further expand the impact of *communication health* in the workforce. Employers can partner with local universities and technical colleges to develop workforce training programs that focus on *communication health* awareness and support skills. This collaboration may include internships and job shadowing opportunities specifically designed for individuals with communication challenges, fostering pathways to sustainable employment. Additionally, public health campaigns may raise awareness among employers about the importance of communication equity, emphasizing how inclusive practices benefit not only employees but also organizational productivity and morale. Businesses may adopt flexible workplace policies that allow for accommodations such as modified communication protocols for employees who use AAC devices. Public health and employment agencies can work together to create guidelines for these policies, offering a framework that companies can adapt to their specific contexts.

### Housing

In housing, policies can address communication needs by incorporating design elements that reduce environmental barriers, such as soundproofing materials in multi-family housing units to minimize noise disruptions. Providing affordable broadband internet access in all housing developments supports telehealth services, online education, and remote work opportunities for individuals relying on virtual communication tools. Local government agencies and housing authorities can also prioritize funding for renovations and upgrades that enhance communication accessibility, particularly in low-income and subsidized housing. Public health agencies can advocate for building codes and standards that promote communication-friendly environments, making certain that accessibility is a core component of housing development rather than an afterthought. Additionally, integrating community-based services into housing complexes may support residents with communication challenges. For instance, creating shared spaces within residential developments where communication support services are available may foster inclusivity.

### Community

Community programs can promote social cohesion and communication equity by offering multilingual workshops, cultural competency training, and resources that facilitate intergenerational dialogue. Libraries present a unique and underutilized opportunity to embed *communication health* promotion and related activities within communities. Libraries receive more visits annually than primary care providers and are widely trusted by the public, making them an ideal setting for supporting *communication health* (46, 47). Given their central role as community hubs, libraries may partner with public health agencies and SLHP+ to offer routine screenings for speech, language, and hearing, especially for children and older adults. These screenings allow for the identification of *communication health* issues early, allowing for timely referrals and intervention. Libraries are also ideal for hosting educational activities that target communication development across the lifespan. Interactive workshops for parents and caregivers can demonstrate strategies for fostering language and literacy at home, while adult-focused programs may include components that address communication skills related to employment and social interactions. In addition to screenings and workshops, libraries can become central venues for public health campaigns aimed at destigmatizing communication disorders (46, 47). By hosting community events that celebrate diverse communication methods such as sign language poetry readings, libraries can promote acceptance while reducing isolation among individuals with communication challenges. Public health professionals can collaborate with librarians to develop and disseminate *communication health* toolkits that include practical resources for caregivers, teachers, community members, and other engagement partners.

## Conclusion

*Communication Public Health* represents the integration of the disciplines related to speech, language, and hearing with public health. It focuses on the prevention of communication disorders and promotion of equality in *communication health* at the population level. Rather than questioning whether speech, language, and hearing health principles are, or should be, part of the field of public health, the focus shifts to integrating the shared principles between the disciplines. This commentary contributes a novel synthesis of speech-language-hearing and public health frameworks, identifying communication health as a critical but overlooked determinant of health. It offers a conceptual foundation for future empirical and policy efforts that more explicitly incorporate communication as a factor in health equity. Embracing this integrated approach allows public health professionals to identify their roles in contributing to health care systems, policy changes, and environments that facilitate optimal communication for all individuals, moving away from a disability-centered lens. The practical value of this framework lies in its potential to influence training, policy, and service delivery models across sectors, not only within healthcare, but in schools, workplaces, and communities. This, in turn, promotes greater *communication health.*

We argue that *communication health* is its own unique entity, distinct from “disability.” Acknowledging *Communication Public Health* as a separate domain allows for targeted interventions and policies aimed at promoting effective communication for all individuals, regardless of ability. By addressing *communication health* directly, initiatives can better target the unique needs and challenges faced by diverse populations. This approach not only enhances health equity but also strengthens social cohesion and fosters a more inclusive society. Therefore, public health efforts must prioritize *communication health* as a fundamental component of overall wellbeing. We advise SLPs, AuDs, SLHP+ and public health professionals to advocate for more formal studies and data-driven evaluations to build a robust evidence base for *Communication Public Health*. Future research should incorporate pilot programs, observational studies and controlled trials to assess improvements in *communication health* and broader health outcomes for individuals who experience speech, language, or hearing disorders. Such empirical efforts would validate the proposed framework and guide the development of interventions and policies to support communication equity.

## Data Availability

The original contributions presented in the study are included in the article/supplementary material, further inquiries can be directed to the corresponding author.

## References

[1] Engel G. The need for a new medical model: a challenge for biomedicine. Science. (1977) 196:129–36. doi: 10.1126/science.847460, PMID: 847460

[2] World Health Organization. Closing the gap in a generation: health equity through action on the social determinants of health – Final report on the commission on social determinants of health. (2008). Available online at: https://www.who.int/publications/i/item/WHO-IER-CSDH-08.1 (Accessed April 28, 2025).

[3] Organization for Economic Co-operation and Development [OECD]. Health at a Glance 2023: OECD Indicators. Paris: OECD Publishing.

[4] Horrigan J. Libraries at the crossroads [internet] pew research center. Internet, Sciences & Tech. (2015). Available online a: http://www.pewinternet.org/2015/09/15/libraries-at-the-42crossraods (Accessed April 28, 2025).

[5] Yorkston KM Baylor CR. Measurement of communicative participation In: Lowitt A Kent R, editors. Assessment of motor speech disorders. San Diego, CA: Plural Publishing (2010). 123–40.

[6] Beard A. Speech, language, and communication: a public health issue across the lifecourse. Paediatr Child Health. (2018) 28:126–31. doi: 10.1016/j.paed.2017.12.004

[7] World Health Organization. World report on hearing. (2021). Available online at: https://www.who.int/publications/i/item/world-report-on-hearing. (Accessed April 28, 2025).

[8] Brandt S Essig S Balthasar A. Professional beliefs of physicians and allied health professionals and their willingness to promote health in primary care: a cross-sectional survey. BMC Prim Care. (2024) 25:188. doi: 10.1186/s12875-024-02412-6, PMID: 38802787 PMC11129482

[9] Comer C Collings R McCracken A Payne C Moore A. Allied health professionals' perceptions of research in the United Kingdom national health service: a survey of research capacity and culture. BMC Health Serv Res. (2002) 22:1094. doi: 10.1186/s12913-022-08465-6, PMID: 36030236 PMC9420271

[10] Enderby P Law J. Speech, language, and communication in a public health context: a UK perspective with potential global application-an opinion piece. Folia Phoniatr Logop. (2019) 71:168–75. doi: 10.1159/000495785, PMID: 31048575

[11] Giusti M Persiani N. Italian health professions of the technical, rehabilitation and prevention areas to support the reform of reference healthcare system after Covid-19 pandemic In. Challenges of healthcare systems in the era of COVID-19: Management practices, services innovation and reforms. Springer Nature Switzerland: Cham (2023). 33–45.

[12] Morris M. Striving toward equity in health care for people with communication disabilities. J Speech Lang Hear Res. (2022) 65:3623–32. doi: 10.1044/2022_JSLHR-22-00057, PMID: 35858270 PMC9802569

[13] Shapiro J. No pity: people with disabilities forging a new civil rights movement. Times Books, New York, NY: Times Books (1994).

[14] Solar O. Irwin A. A conceptual framework for action on the social determinants of health. Social Determinants of Health Discussion Paper 2 (Policy and Practice), World Health Organization. (2010). Available online at: https://iris.who.int/handle/10665/44489 (Accessed April 28, 2025).

[15] Threats T. Towards an international framework for communication disorders: use of the ICF. J Commun Disord. (2006) 39:251–65. doi: 10.1016/j.jcomdis.2006.02.002, PMID: 16597447

[16] Warren S Lopez L Anthony T Coco L. Communication public health: an integration of audiology, speech-language pathology, and public health. J Speech Lang Hear Res. (2024) 67:1–18. doi: 10.1044/2024_JSLHR-23--00491 39083459

[17] American Speech-Language-Hearing Association. Scope of practice in speech-language pathology. Available online at: https://www.asha.org/policy/sp2016–00343/ (Accessed July 15, 2024).

[18] American Speech-Language-Hearing Association. Scope of practice in audiology. Available online at: https://www.asha.org/policy/sp2018-00353/ (Accessed July 15, 2024).

[19] Giusti M Persiani N. Art therapy in Alzheimer's disease: an opportunity of collaboration between intersectoral public and private organizations in the co-design of health and social care services. Front Psych. (2023) 14:1198613. doi: 10.3389/fpsyt.2023.1198613 PMC10720310 38098624

[20] Royal College of Speech & Language Therapists. Communication access UK: inclusive communication for all. Available online at: https://www.rcslt.org/policy-and-influencing/communication-access-uk/#:~:text=Up%20to%2014%20million%20people,a%20long%2Dterm%20communication%20need (Accessed December 2, 2024).

[21] Australian Bureau of Statistics. Disability, ageing and carers, Australia: summary of findings. Available online at: https://www.abs.gov.au/AUSSTATS/abs@.nsf/Lookup/4430.0Main+Features872015?OpenDocument#:~:text=Back%20to%20top-,Level%20of%20communication%20disability,differences%20were%20not%20statistically%20significant (Accessed December 2, 2024).

[22] National Center for Health Statistics. Percentage of any difficulty communicating for adults aged 18 and over, United States, 2019–2022. National Health Interview Survey. (2023). Available online at: https://www.cdc.gov//NHISDataQueryTool/SHS_adult/index.html (Accessed July 10, 2024).

[23] National Institute on Deafness and Other Communication Disorders [NIDCD]. Statistics and epidemiology. (2025) Available online at: https://www.nidcd.nih.gov/health/statistics (Accessed July 10, 2024).

[24] National Academies of Sciences, Engineering, and Medicine. Hearing health care for adults: priorities for improving access and affordability. Washington, DC: The National Academies Press (2016). 27280276

[25] Dimer N Rech R Chiari B Goulart B. Prevalence of speech-language and hearing disorders in elderly and younger adults according to sex and age: a population survey. CoDAS. (2023) 33:e20200080. doi: 10.1590/2317-1782/20202020080 34133611

[26] Di Sante M Potvin L. We need to talk about social inequalities in language development. Am J Speech Lang Pathol. (2022) 31:1894–7. doi: 10.1044/2022_AJSLP-21-00326, PMID: 35442715

[27] Doll E Braden M Thibeault S. COVID-19 and speech-language pathology clinical practice of voice and upper airway disorders. Am J Speech Lang Patholo. (2021) 30:63–74. doi: 10.1044/2020_AJSLP-20-00228, PMID: 33332145 PMC8740584

[28] Schuh M Bush M. Defining disparities in cochlear implantation through the social determinants of health. Semin Hear. (2021) 42:321–30. doi: 10.1055/s-0041-1739282, PMID: 34912160 PMC8660167

[29] Schuh M Bush M. Evaluating equity through the social determinants of hearing health. Ear Hear. (2022) 43:15S–22S. doi: 10.1097/AUD.0000000000001188, PMID: 35724251 PMC9219021

[30] Haahr A Groos H Sorensen D. ‘Striving for normality’ when coping with Parkinson’s disease in everyday life: a metasynthesis. Int J Nurs Stud. (2021) 118:103923. doi: 10.1016/j.ijnurstu.2021.103923, PMID: 33813086

[31] Office of Disease Prevention and Health Promotion. Social determinants of health. Health People 2030. U.S. Department of Health and Human Services. (2025) Available online at: https://health.gov/healthypeople/objectives-and-data/social-determinants-health (Accessed July 10, 2024).

[32] Carragher M Steel G O’Halloran R Lamborn E Torabi T Johnson H. Aphasia disrupts usual care: “I’m not mad, I’m not deaf” – the experiences of individuals with aphasia and family members in hospital. Disabil Rehabil. (2024) 46:6122–33. doi: 10.1080/09638288.2024.2324115, PMID: 38444182

[33] International Association of Communication Sciences and Disorders [IALP]. Position paper on speech-language pathology services in low-and middle-income countries. (2018). Available online at: https://ialpasoc.info/resources (Accessed April 28, 2025).

[34] Farmanova E Bonneville L Bouchard L. Organizational health literacy: review of theories, frameworks, guides, and implementation issues. Inquiry. (2018) 55:0046958018757848. doi: 10.1177/0046958018757848 29569968 PMC5871044

[35] Williams B Fuller T Jurturu P Rashoka F Vasquez J Oluwemimo D. Words matter: the languages of public health. Am J Public Health. (2024) 114:140–1. doi: 10.2105/AJPH.2023.307540, PMID: 38335495 PMC10862212

[36] Rashoka F Vasquez J Williams B Fuller T Juturu P Oluwemimo D. The languages of public health: student perspectives on terms, communities, and languages. Am J Public Health. (2024) 114:164–5. doi: 10.2105/AJPH.2023.307545, PMID: 38335489 PMC10862222

[37] Guiffre S Domholdt E Keehan J. Beyond the individual: population health and physical therapy. Physiother Theory Pract. (2020) 36:564–71. doi: 10.1080/09593985.2018.1490364 30019979

[38] Zughni L Gillespie A Hatcher J Rubin A Giliberto J. Telemedicine and the interdisciplinary clinic model: during the COVID-19 pandemic and beyond. Otolaryngol Head Neck Surg. (2020) 163:673–5. doi: 10.1177/0194599820932167, PMID: 32484731

[39] Glanz K Bishop D. The role of behavioral science theory in development and implementation of public health interventions. Annu Rev Public Health. (2010) 31:399–418. doi: 10.1146/annurev.publhealth.012809.103604 20070207

[40] Ayoub M Larrouy-Maestri P Morsomme D. The effect of smoking on the fundamental frequency of the speaking voice. J Voice. (2019) 33:802.e11–6. doi: 10.1016/j.jvoice.2018.04.001, PMID: 29748027

[41] Snow P. Speech-language pathology and the youth offender: epidemiological overview and roadmap for future speech-language pathology research and scope of practice. Lang Speech Hear Serv Sch. (2019) 50:324–39. doi: 10.1044/2018_LSHSS-CCJS-18-0027, PMID: 31017853

[42] Lawrence M Kerr S Watson H Paton G Ellis G. An exploration of lifestyle beliefs and lifestyle behaviour following stroke: findings from a focus group study of patients and family members. BMC Fam Pract. (2010) 11:Article 97. doi: 10.1186/1471-2296-11-97, PMID: 21143874 PMC3018456

[43] Kroger A. Bahta L. Long S. Sanchez P. General best practice guidelines for immunization. (2024) Available online at: https://www.cdc.gov/vaccines/hcp/acip-recs/general-recs/downloads/general-recs.pdf (Accessed July 15, 2024).

[44] Hou Y Zhou A Brooks L Reid D Turkstra L MacDonald S. Rehabilitation access for individuals with cognitive-communication challenges after traumatic brain injury: a co-design study with person with lived experience. Int J Lang Commun Disord. (2024) 59:648–64. doi: 10.1111/1460-6984.12895, PMID: 37189286

[45] Xia W Yan H Zhang Y Wang C Gao W Lv C. Congenital human cytomegalovirus infection introducing sensorineural hearing loss. Front Microbiol. (2021) 12:649690. doi: 10.3389/fmicb.2021.649690, PMID: 33936007 PMC8079719

[46] Hornik R. Public health communication. Abingdon: Lawrence Erlbaum Associates (2002).

[47] Horrigan J. Libraries [internet] pew research center: internet. Science & Tech (2016). Available online at: http://www.pewinternet.org/2016/09/09/libraries-2016/

[48] Giusti M Nardi C Bonaccorsi G Lorini C Persiani N. Organizational health literacy as a supportive tool for the effective implementation of the 2013/59/EURATOM directive in Italy. Ann Istit Supper San. (2024) 60:145–53. doi: 10.4415/ANN_24_02_09, PMID: 38984629

